# Pattern‐Based P53 Immunohistochemistry Predicts Recurrence in Colorectal Liver Metastases Without Complete Pathologic Response After Neoadjuvant Chemotherapy

**DOI:** 10.1002/cnr2.70701

**Published:** 2026-10-03

**Authors:** Phat Thi Hong Ho, Duy Duc Nguyen, Vuong Dinh Thy Hao, Giang Huong Tran, Lam Viet Trung, Thinh Van Hoang, Hoang Bac Nguyen

**Affiliations:** ^1^ Department of Pathology University Medical Center Ho Chi Minh City Ho Chi Minh City Vietnam; ^2^ Department of Medical Oncology, Oncology Center—Cho Ray Hospital Ho Chi Minh City Vietnam; ^3^ Department of Histology, Embryology and Pathology, School of Medicine University of Medicine and Pharmacy at Ho Chi Minh City Ho Chi Minh City Vietnam; ^4^ Digestive Surgery Department Cho Ray Hospital Ho Chi Minh City Vietnam; ^5^ Department of Pathology Cho Ray Hospital Ho Chi Minh City Vietnam; ^6^ Department of General Surgery, School of Medicine University of Medicine and Pharmacy at Ho Chi Minh City Ho Chi Minh City Vietnam; ^7^ University Medical Center Ho Chi Minh City Ho Chi Minh City Vietnam

**Keywords:** colorectal liver metastases, p53, recurrence‐free survival

## Abstract

**Background:**

Aberrant p53 expression may have prognostic significance in colorectal cancer; however, its role in patients with colorectal liver metastases (CRLM) who receive neoadjuvant chemotherapy but do not achieve pathological complete response (pCR) remains unclear.

**Aims:**

We evaluated whether aberrant p53 expression assessed by immunohistochemistry (IHC) predicts recurrence‐free survival (RFS) in patients with CRLM receiving neoadjuvant FOLFOXIRI followed by resection without achieving pCR.

**Methods and Results:**

A prospective cohort study was conducted on 39 patients with CRLM who met the inclusion criteria. All patients received neoadjuvant chemotherapy with the FOLFOXIRI regimen. Following surgical resection of both primary colorectal tumors and liver metastases, none of the patients achieved pCR. Immunohistochemistry was performed to assess p53 expression in resected liver metastasis specimens using a pattern‐based interpretation model. RFS was analyzed using univariate and multivariate Cox proportional hazards models. Aberrant p53 expression was observed in 20 patients (51.3%). In univariate analysis, aberrant p53 expression (HR = 2.20; 95% CI: 1.02–4.71; *p* = 0.043), total tumor size > 6 cm (HR = 5.62; 95% CI: 2.31–13.68; *p* < 0.001), number of liver metastases > 4 (HR = 4.41; 95% CI: 1.59–12.26; *p* = 0.004), and positive surgical margin (HR = 2.62; 95% CI: 1.16–5.91; *p* = 0.021) were significantly associated with recurrence. In multivariate analysis, aberrant p53 expression (HR = 3.78; 95% CI: 1.56–9.15; *p* = 0.003) and total tumor size > 6 cm (HR = 5.23; 95% CI: 1.56–17.55; *p* = 0.007) remained independent prognostic factors for shorter RFS.cnr270701_RedLinePDF.

**Conclusion:**

Aberrant p53 expression, as assessed by immunohistochemistry on post‐neoadjuvant chemotherapy specimens, is an independent prognostic factor for early recurrence in patients with CRLM who did not achieve pCR. Together with total tumor size, aberrant p53 expression may serve as a useful marker for risk stratification and personalized postoperative treatment strategies in this high‐risk patient population.

## 1 Introduction

Colorectal cancer (CRC) is among the most common malignancies worldwide and remains a leading cause of cancer‐related mortality. According to the GLOBOCAN 2020 estimates, CRC ranks third in incidence and second in mortality, with over 1.9 million new cases and nearly 935 000 deaths annually [1]. Despite significant advancements in screening and treatment strategies, a substantial proportion of patients are still diagnosed at an advanced stage or with metastatic disease, imposing a considerable burden on healthcare systems and society [1, 2]. Among the metastatic sites, the liver is the most commonly affected organ due to the portal venous system, which drains blood directly from the colorectal region to the liver. At the time of diagnosis, approximately 13.5% of CRC cases present with colorectal liver metastases (CRLM) [3]. In patients eligible for surgery, the combination of neoadjuvant chemotherapy and hepatic resection can significantly improve treatment outcomes by increasing the likelihood of complete (R0) resection and reducing the risk of postoperative recurrence [4].

One of the key indicators of treatment efficacy is the achievement of a pathologic complete response (pCR) in the liver, defined as the absence of viable cancer cells in metastatic tissue following chemotherapy. However, previous studies have shown that only approximately 4%–6% of patients with CRLM achieve pCR, while the majority do not, and these patients are associated with significantly worse prognoses [5, 6]. Numerous studies have demonstrated that patients who achieve pCR exhibit higher overall survival rates, longer recurrence‐free survival, and lower recurrence rates compared to those who do not achieve pCR [6, 7, 8]. In addition to clinical and prognostic differences, these two groups may also differ in histopathological and molecular characteristics, such as RAS or BRAF mutations, or the expression of proteins associated with mechanisms of therapeutic resistance [9, 10].

Among the molecular markers in CRC, p53—the protein product of the TP53 gene—plays a central role in regulating the cell cycle, facilitating DNA repair, and apoptosis [11]. TP53 mutation is one of the most common genetic alterations in CRC and may lead to dysregulated cell proliferation and resistance to chemotherapy [12, 13]. Assessment of p53 expression by immunohistochemistry (IHC) is a simple, feasible, and widely adopted method in routine pathological practice. Several studies have suggested that aberrant p53 expression may be associated with poor prognosis in patients with CRC [14, 15]. However, the results remain inconsistent, partly due to variations in the criteria used to evaluate p53 expression across studies. Some earlier quantitative standards, such as the 10% cut‐off, have been shown to inaccurately reflect TP53 mutation status [14, 15, 16]. Recently, Köbel et al. proposed a pattern‐based classification model for aberrant p53 expression based on studies in ovarian carcinoma, and demonstrated that this approach has high accuracy in predicting TP53 mutations [16]. Therefore, applying this classification standard to assess p53 expression and evaluating its prognostic value in CRC, particularly in the CRLM subgroup, is necessary to standardize interpretation and enhance the clinical utility of p53 IHC testing.

However, there remains a lack of dedicated studies on the prognostic role of p53 expression in patients with CRLM who do not achieve pCR, a majority subgroup characterized by a high risk of recurrence. In particular, the application of the newly defined aberrant p53 pattern by IHC has not been fully validated in this context. This gap highlights the need to re‐evaluate the prognostic potential of p53 as a biomarker to facilitate risk stratification and personalized treatment in patients with poor prognosis. Therefore, this study was conducted to investigate the association between p53 expression (as assessed by IHC) and recurrence‐free survival (RFS) in CRC patients with liver metastases who underwent neoadjuvant chemotherapy but did not achieve pCR.

## 2 Materials and Methods

### 2.1 Study Population and Eligibility Criteria

A prospective cohort study was conducted at Cho Ray Hospital from January 2022 to June 2023, enrolling 92 consecutive patients diagnosed with colorectal liver metastases (CRLM). All patients received neoadjuvant chemotherapy with the FOLFOXIRI regimen, administered intravenously every 2 weeks. Treatment responses were uniformly assessed every 8–9 weeks by the same multidisciplinary tumor board using predefined surgical eligibility criteria.

Of the 92 patients, 54 were deemed eligible for surgical resection of both the primary colorectal tumor and liver metastases according to these standardized criteria. The subsequent reduction to 39 patients was not due to selective sampling but reflected the routine clinical treatment pathway. Among the 54 resected cases, four patients were excluded due to inadequate paraffin block quality for immunohistochemical analysis, 10 achieved pathological complete response (pCR) on postoperative histopathology (and were therefore outside the scope of this study), and one was determined to be unresectable intraoperatively. Ultimately, 39 patients with residual viable tumor after neoadjuvant chemotherapy were included in the final analysis (Figure 1).

**FIGURE 1:**
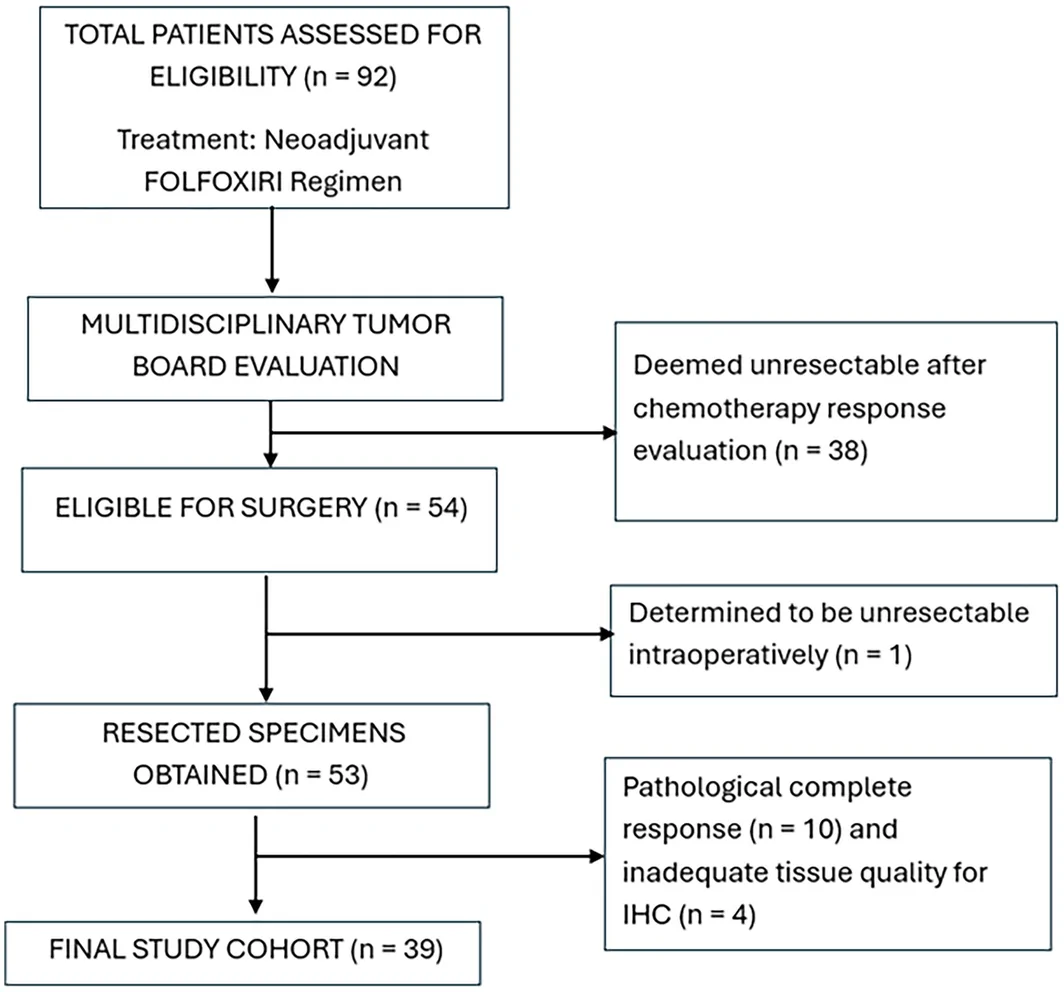
Patient selection flow diagram.

Following surgery, patients were stratified according to hepatic resection margin status:

1. Patients who underwent R0 resection (complete removal of all macroscopic disease) proceeded to complete a total of 12 cycles of FOLFOXIRI. These patients were subsequently monitored at scheduled intervals (every 3 months in the first year and every 4 months in the second year) using imaging studies (CT or MRI) and serum carcinoembryonic antigen (CEA) levels. Disease status (progression, stable disease, or recurrence) was determined based on the Response Evaluation Criteria in Solid Tumors (RECIST) version 1.1 and serum CEA levels, evaluated by the same oncology specialist throughout the follow‐up period.
2. Patients with R1 or R2 resection (microscopic or gross residual disease) also completed 12 cycles of FOLFOXIRI, with additional local ablative treatments such as radiofrequency ablation (RFA) or microwave ablation (MWA) as appropriate. Maintenance chemotherapy with either 5‐FU/leucovorin or capecitabine was continued until disease progression. Disease status (progression, stable disease, or recurrence) was determined based on imaging findings and serum CEA levels, and was evaluated by the same oncology specialist throughout follow‐up.

Patient inclusion criteria were: (1) Histologically confirmed CRLM with liver resection performed at Cho Ray Hospital during the study period; (2) presence of residual viable tumor cells postneoadjuvant chemotherapy (non‐pCR); (3) availability of well‐preserved tissue sections and paraffin blocks suitable for IHC analysis; and (4) age between 18 and 75 years with an ECOG performance status of 0 or 1 at the time of surgery. Exclusion criteria included: (1) Recurrent disease identified at the time of surgery; and (2) patients who had received chemotherapy prior to their initial evaluation at Cho Ray Hospital.

### 2.2 Study Variables

Clinical and laboratory variables collected included age, sex, preoperative serum carcinoembryonic antigen (CEA) levels, HBV/HCV infection status, and number of chemotherapy cycles.

Histopathologic features were assessed using resected liver metastasis specimens and included: number and size of metastatic lesions, degree of tumor‐infiltrating lymphocytes (TILs), resection margin status (positive or negative), background liver pathology (presence of cirrhosis, hepatitis grade based on the METAVIR score, and steatosis), and percentage of residual tumor cells following chemotherapy. Patients who achieved pCR were excluded from histopathologic analysis.

Recurrence‐free survival was defined as the interval from the date of liver resection (R0 or R1) to the occurrence of tumor recurrence (either locoregional or distant) or death attributable to tumor progression. Deaths unrelated to tumor pathology were not considered events in the RFS analysis. Survival time was measured in months.

### 2.3 Molecular Analysis of KRAS, NRAS, and BRAF

Among the 39 included patients, results of KRAS, NRAS, and BRAF mutation testing were available for 38 cases. Mutation analysis was performed in the Department of Pathology, Cho Ray Hospital, using pyrosequencing technique on a Qiagen platform, according to the manufacturer's instructions. Because of the limited sample size, KRAS, NRAS, and BRAF mutations were grouped into a single composite variable, termed RAS/BRAF alterations, for exploratory analysis.

### 2.4 Evaluation of p53 Expression by Immunohistochemistry

Immunohistochemical staining for p53 was performed on liver metastasis specimens using a monoclonal anti‐p53 antibody (clone DO‐7, Dako). Staining was carried out automatically according to the manufacturer's instructions on the BenchMark XT platform (Ventana). p53 expression was evaluated using a pattern‐based interpretation model, as described by Köbel et al. Aberrant p53 expression was defined as either: (1) overexpression if strong and diffuse nuclear staining in ≥ 80% of tumor cells; or (2) complete absence of expression if no detectable nuclear staining in tumor cells. Cases not meeting these criteria were classified as having a wild‐type expression pattern [16].

### 2.5 Statistical Analysis

Data were analyzed using SPSS software, version 27. Categorical variables were compared using the chi‐squared test or Fisher's exact test, as appropriate. Univariate and multivariate Cox proportional hazards regression analyses were employed to assess the prognostic impact of variables associated with tumor recurrence.

An additional exploratory univariate Cox analysis was performed for patients with concurrent aberrant p53 expression and RAS/BRAF alterations.

Given the limited sample size (*n* = 39), to minimize overfitting in the multivariate Cox model, only four variables were included: p53 expression and the three additional variables with the lowest *p*‐values in univariate analysis. This selection strategy was implemented to maintain an appropriate ratio between the number of events and the number of covariates, in accordance with established recommendations for regression modeling. A two‐sided *p*‐value < 0.05 was considered statistically significant.

## 3 Results

This study included 39 patients, with a mean age of 56.36 ± 10.15 years. The male‐to‐female ratio was 29 (74.36%) to 10 (25.64%). Among these, 19 patients (48.7%) exhibited wild‐type p53 expression, while 20 patients (51.3%) showed aberrant p53 expression. At the time of analysis, the median RFS was 9.4 months (95% CI: 6.2–12.1), with a total of 35 recurrence events observed during the follow‐up period.

### 3.1 Association Between p53 Expression and Clinical, Laboratory, and Histopathologic Characteristics

Aberrant p53 expression appeared to be more common among patients with elevated preoperative CEA levels (> 5 U/L) and higher ECOG performance status (score of 1); however, these associations were not statistically significant (*p* = 0.811 and *p* = 0.958, respectively).

ECOG performance status 1 was more frequent in the aberrant p53 group than in the wild‐type group (*p* = 0.029). No other clinical or histopathologic variables showed significant differences between the two groups (Tables 1 and 2).

**TABLE 1:** Association between clinical and paraclinical characteristics, p53 expression, and risk of tumor recurrence.

Factors	p53 expression pattern			*p* (Groups)	Tumor recurrence HR (95% CI)	*p* (Cox)
	Total (*n* = 39)	Wild‐type (*n* = 19)	Aberrant (*n* = 20)			
Age (*n* = 39)						
< 60	22 (56.41)	11 (50.00)	11 (50.00)		1	
≥ 60	17 (43.59)	8 (47.06)	9 (52.94)	0.863^a^	0.60 (0.28–1.30)	0.195
Sex (*n* = 39)						
Male	29 (74.36)	13 (44.83)	16 (55.17)		1	
Female	10 (25.64)	6 (60.00)	4 (40.00)	0.480^b^	0.69 (0.28–1.71)	0.428
CEA (U/L) (*n* = 38)						
≤ 5	4 (10.53)	4 (100)	0 (0)		1	
> 5	34 (89.47)	15 (44.12)	19 (55.88)	0.105^b^	1.16 (0.35–3.90)	0.811
Depth of tumor response (*n* = 39)						
< 30%	3 (7.69)	3 (100)	0 (0)		1	
≥ 30%	36 (92.31)	16 (44.44)	20 (55.56)	0.106^b^	0.36 (0.10–1.26)	0.109
KRAS/NRAS/BRAF mutations (*n* = 39)						
Wild‐type	24 (61.54)	11 (45.83)	13 (54.17)		1	
Mutated	15 (38.46)	8 (53.33)	7 (46.67)	0.647^a^	1.53 (0.71–3.31)	0.281
Microsatellite instability (*n* = 39)						
No	39 (100)	19 (48.72)	20 (51.28)			
Yes	0 (0.00)	0 (0.00)	0 (0.00)	1^b^		
Body mass index^d^ (*n* = 39)	22.69 (3.19)	22.92 (3.81)	22.48 (2.56)	0.667^c^	1.05 (0.95–1.17)	0.309
ECOG performance status (*n* = 39)						
ECOG 0	24 (61.54)	15 (62.50)	9 (37.50)		1	
ECOG 1	15 (38.46)	4 (26.67)	11 (73.33)	0.029^a^	0.98 (0.47–2.03)	0.958
HBV (*n* = 39)						
Negative	34 (87.18)	16 (47.06)	18 (52.94)		1	
Positive	5 (12.82)	3 (60.00)	2 (40.00)	0.661^b^	0.46 (0.11–1.95)	0.290
HCV (*n* = 39)						
Negative	38 (97.44)	18 (47.37)	20 (52.63)		1	
Positive	1 (2.56)	1 (100)	0 (0)	0.487^b^		
*RAS/BRAF* alterations (*n* = 38)^e^						
Wild‐type	23 (60.53)	11 (47.83)	12 (52.17)		1	
Mutation	15 (39.47)	8 (53.33)	7 (46.67)	0.740^a^	1.62 (0.73–3.60)	0.232
Preoperative chemotherapy (cycles) (*n* = 39)						
12 cycles	19 (48.72)	9 (47.37)	10 (52.63)		1	
< 12 cycles	20 (51.28)	10 (50.00)	10 (50.00)	0.862^a^	0.34 (0.15–0.74)	0.007

Abbreviations: BMI, body mass index; BRAF, B‐Raf Proto‐Oncogene, Serine/Threonine Kinase; CEA, carcinoembryonic antigen; CI, confidence interval; ECOG, Eastern cooperative oncology group; HBV, hepatitis B virus; HCV, hepatitis C virus; KRAS, Kirsten Rat Sarcoma viral oncogene homolog; NRAS, neuroblastoma RAS viral oncogene homolog.

^a^ Chi‐squared test.

^b^ Fisher's exact test.

^c^ Independent samples *t*‐test.

^d^ Reported as mean ± standard deviation.

^e^ One case was excluded due to missing data.

**TABLE 2:** Association between histopathologic features of liver metastases, p53 expression, and risk of tumor recurrence.

Factors	p53 expression pattern			*p* (Groups)	Tumor recurrence HR (95% CI)	*p* (Cox)
	Total (*n* = 39)	Wild‐type (*n* = 19)	Aberrant (*n* = 20)			
Largest tumor size (*n* = 39)						
≤ 2 cm	19 (48.72)	7 (36.84)	12 (63.16)		1	
> 2 cm	20 (51.28)	12 (60.00)	8 (40.00)	0.148^a^	1.49 (0.71–3.12)	0.293
Total tumor size (*n* = 39)						
≤ 6 cm	27 (69.23)	13 (48.15)	14 (51.85)		1	
> 6 cm	12 (30.77)	6 (50.00)	6 (50.00)	0.920^a^	5.62 (2.31–13.68)	< 0.001
Number of lesions (*n* = 39)						
≤ 4	34 (87.18)	17 (50.00)	17 (50.00)		1	
> 4	5 (12.82)	2 (40.00)	3 (60.00)	1^b^	4.41 (1.59–12.26)	0.004
Residual tumor cell percentage (*n* = 39)						
< 70%	23 (58.97)	11 (47.83)	12 (52.17)		1	
≥ 70%	16 (41.03)	8 (50.00)	8 (50.00)	0.888^a^	1.52 (0.70–3.31)	0.292
TILs (*n* = 39)						
≤ 10%	20 (51.28)	9 (45.00)	11 (55.00)		1	
> 10%	19 (48.72)	10 (52.63)	9 (47.37)	0.632^a^	1.24 (0.58–2.66)	0.582
Resection margin status (*n* = 39)						
Negative	26 (66.67)	13 (50.00)	13 (50.00)		1	
Positive	13 (33.33)	6 (46.15)	7 (53.85)	0.823^a^	2.62 (1.16–5.91)	0.021
Liver cirrhosis (*n* = 39)						
No	35 (89.74)	17 (48.57)	18 (51.43)		1	
Yes	4 (10.26)	2 (50.00)	2 (50.00)	1^b^	1.80 (0.61–5.36)	0.287
METAVIR inflammation score (*n* = 39)						
≤ 2 score	27 (69.23)	13 (48.15)	14 (51.85)		1	
3 score	12 (30.77)	6 (50.00)	6 (50.00)	0.920^a^	0.87 (0.40–1.89)	0.716
Hepatic steatosis (*n* = 39)						
None–mild	34 (87.18)	16 (47.06)	18 (52.94)		1	
Moderate–severe	5 (12.82)	3 (60.00)	2 (40.00)	0.661^b^	0.89 (0.35–2.25)	0.800

Abbreviation: TILs, tumor‐infiltrating lymphocytes.

^a^ Chi‐squared test.

^b^ Fisher's exact test.

### 3.2 Association Between p53 Expression and Clinicopathologic Features in Relation to Recurrence Risk

In patients with aberrant p53 expression, the cumulative recurrence rate increased more rapidly and reached 100% by 14 months postoperatively. Specifically, at 6 months, the estimated recurrence probability in this group was 46.4% (95% CI: 27.3%–70.5%), compared to 21.4% (95% CI: 8.6%–47.5%) in the wild‐type p53 group. By month 19, the recurrence rate in the wild‐type group rose to 83.3% (95% CI: 59.8%–97.0%), whereas the aberrant group had already reached 100% prior to that point (Figure 2).

**FIGURE 2:**
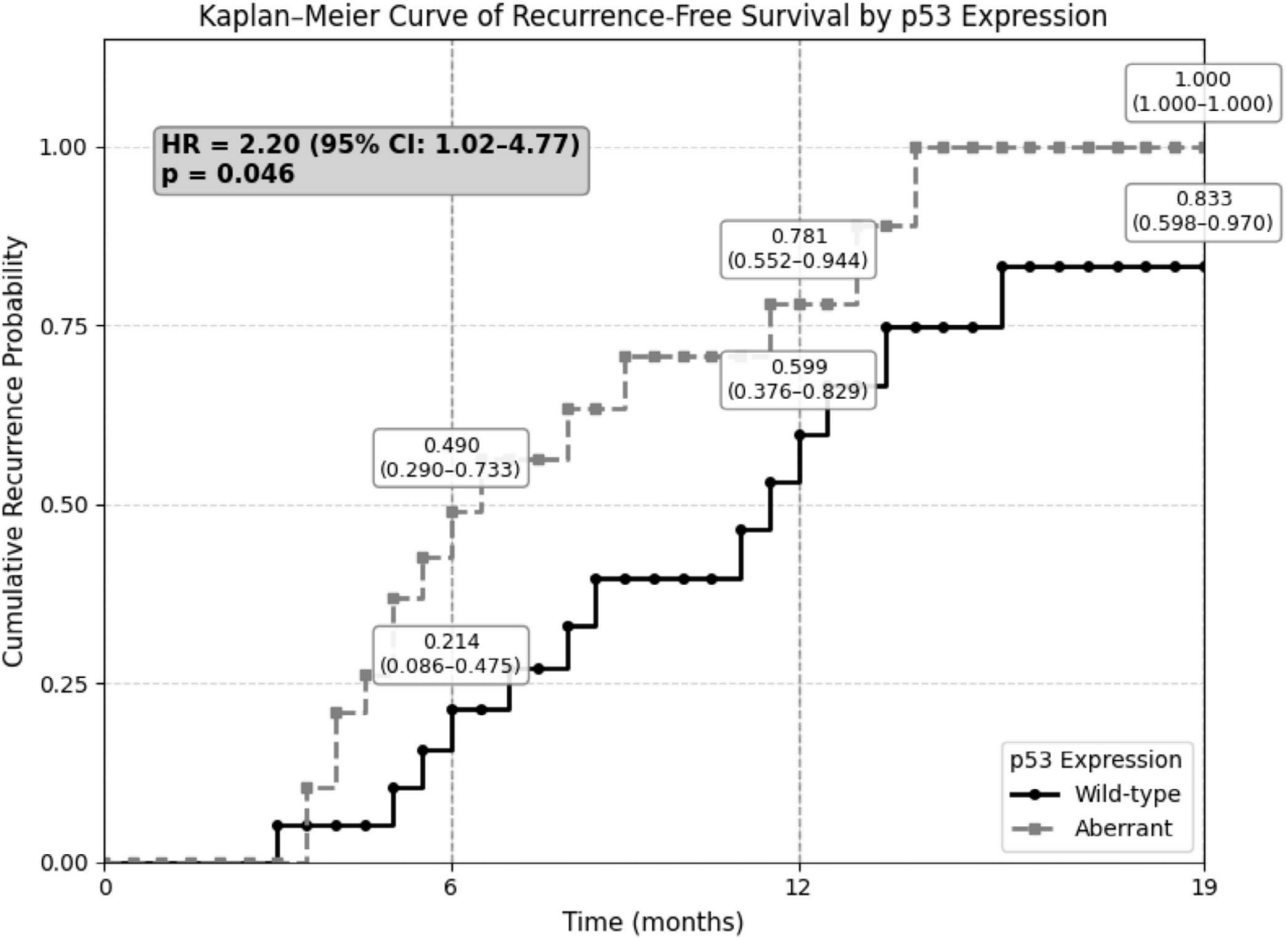
Kaplan–Meier curve of recurrence‐free survival stratified by p53 expression pattern.

In addition, completion of the full 12 cycles of neoadjuvant chemotherapy prior to surgery was significantly associated with a reduced risk of recurrence (HR = 0.34, 95% CI: 0.15–0.74; *p* = 0.007) (Table 1). Several histopathologic features also showed strong associations with RFS, among which a total tumor size > 6 cm demonstrated the highest recurrence risk (HR = 5.62, 95% CI: 2.31–13.68; *p* < 0.001). Other factors associated with increased recurrence included the presence of more than four liver metastases (HR = 4.41, 95% CI: 1.59–12.26; *p* = 0.004) and a positive resection margin (HR = 2.62, 95% CI: 1.16–5.91; *p* = 0.021) (Table 2).

Multivariate analysis was performed using the four variables with the lowest *p*‐values from univariate analysis: p53 expression, total tumor size, number of liver metastases, and number of preoperative chemotherapy cycles. The results showed that aberrant p53 expression remained an independent prognostic factor, associated with a 3.78‐fold increased risk of recurrence compared to wild‐type expression (HR = 3.78, 95% CI: 1.56–9.15; *p* = 0.003). Similarly, a total tumor size > 6 cm retained its prognostic significance (HR = 5.23, 95% CI: 1.56–17.55; *p* = 0.007). In contrast, the number of liver metastases > 4 and receiving fewer than 12 chemotherapy cycles prior to surgery were no longer statistically significant in the multivariate model (*p* = 0.968 and *p* = 0.110, respectively), suggesting limited prognostic value after adjustment for other covariates (Figure 3).

**FIGURE 3:**
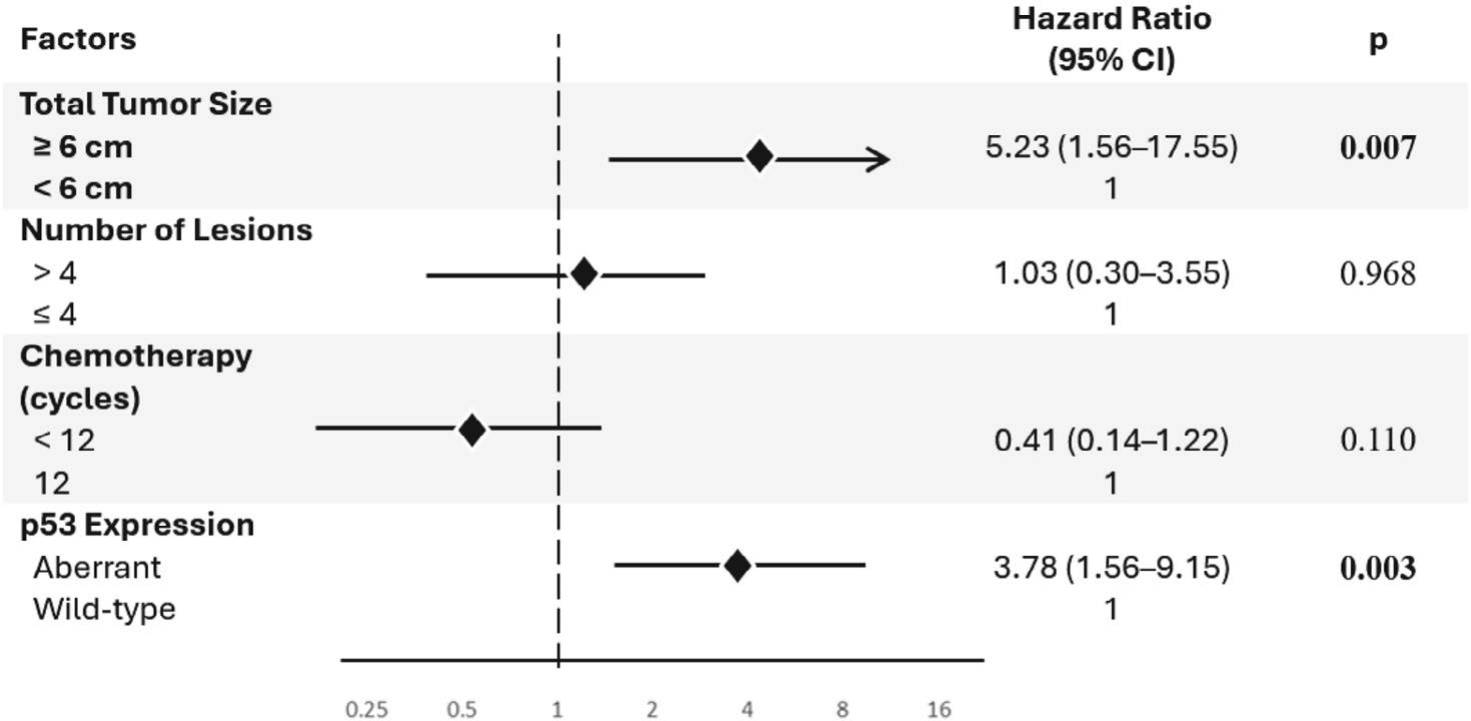
Multivariate analysis of prognostic factors associated with recurrence‐free survival.

Overall survival (OS) analysis was additionally performed. In univariate Cox analysis, p53 expression was not associated with OS (HR = 0.73, 95% CI: 0.16–3.27; *p* = 0.678).

Molecular data for KRAS, NRAS, and BRAF were available in 38 patients. In exploratory analysis, these alterations were grouped as RAS/BRAF alterations. No significant association was observed between concurrent aberrant p53 expression and RAS/BRAF alterations and recurrence‐free survival (HR = 0.49, 95% CI: 0.09–2.60; *p* = 0.400).

## 4 Discussion

Our study was conducted on a cohort of 39 patients with CRLM who underwent neoadjuvant chemotherapy but did not achieve a pathologic complete response (pCR). Aberrant p53 expression was observed in 51.3% of cases. Both aberrant p53 expression and a total metastatic tumor size greater than 6 cm were identified as independent prognostic factors associated with an increased risk of early postoperative recurrence.

While the prognostic role of p53 in primary colorectal cancer has been demonstrated in numerous studies, its significance in CRLM remains controversial in the literature [17, 18]. This uncertainty arises not only from the heterogeneity of study populations and the complex interactions between p53 and other genetic alterations in CRC, but also from inconsistencies in the definition of p53 expression by immunohistochemistry (IHC) across studies. A systematic review by Toren W reported that positive p53 expression was observed in approximately 65% of CRLM cases, similar to our findings [17]. Several early studies from the 1990s by Nitti et al. and Belluco et al. reported that positive p53 expression was a prominent adverse prognostic marker in patients with liver metastases. However, these studies employed a 10% cut‐off to define p53 positivity and were not conducted in patient cohorts treated with neoadjuvant chemotherapy, which distinguishes them from our study [19, 20]. Another study by Molleví et al. [15] identified TP53 mutation as an adverse prognostic factor in patients with CRLM (HR = 1.80, 95% CI: 1.07–3.06; *p* = 0.028). The study also reported a poor correlation between TP53 mutation status and p53 immunohistochemical expression when using a 10% cut‐off value, with a Kappa coefficient of 0.12 (*p* = 0.25) [15]. In contrast, several studies have failed to demonstrate a significant association between p53 expression and disease progression. A study by de Jong et al. [21], which analyzed 44 surgically treated CRLM patients, concluded that p53 expression status had no impact on 5‐year overall survival in this cohort (*p* = 0.9). Notably, this study also adopted the same p53 IHC cut‐off definition as earlier investigations [21]. A study by Pilat et al. employed molecular techniques to detect TP53 mutations in 51 CRLM patients who received neoadjuvant chemotherapy and 25 patients who did not. The results showed that TP53 mutation was a significant prognostic factor in the chemotherapy group (HR = 3.12, 95% CI: 1.46–6.95; *p* = 0.0025), consistent with our findings. In contrast, no significant survival difference was observed in the nonchemotherapy group (HR = 0.66, 95% CI: 0.19–3.04; *p* = 0.54) [22]. A more recent study by Kim et al. [14], utilizing pattern‐based interpretation criteria, demonstrated an 80.4% concordance between p53 immunohistochemical expression and TP53 mutation status. Moreover, the study showed that aberrant p53 expression detected by IHC had greater prognostic accuracy for overall survival than TP53 mutations identified by next‐generation sequencing (NGS), with hazard ratios of 4.352 (95% CI: 1.288–14.712; *p* = 0.018) and 1.739 (95% CI: 0.588–5.142; *p* = 0.317), respectively [14]. Taken together, recent evidence, particularly from studies involving patients who received neoadjuvant chemotherapy or employed the updated pattern‐based criteria for p53 evaluation, suggests that aberrant p53 expression holds significant potential as a prognostic marker in CRLM patients following neoadjuvant treatment.

From a molecular standpoint, aberrant p53 expression reflects a loss of tumor‐suppressor activity, leading to dysregulation of cell cycle control and apoptosis. This dysfunction has been implicated in several biological processes that may underlie early recurrence. First, p53 inactivation can upregulate matrix metalloproteinase (MMP) activity, thereby facilitating tumor invasion and metastatic spread [23, 24]. Second, it may promote epithelial–mesenchymal transition (EMT), enhancing the migratory and invasive capacities of tumor cells [25]. Third, loss of p53 function has been shown to alter the tumor microenvironment, promoting angiogenesis and impairing host antitumor immune responses [26, 27]. Finally, aberrant p53 expression contributes to chemoresistance. Many commonly used neoadjuvant agents in colorectal cancer, including oxaliplatin and irinotecan (components of the FOLFOXIRI regimen), exert their cytotoxic effects by inducing DNA damage and activating p53‐dependent apoptotic pathways [13, 28]. When p53 is mutated or dysfunctional, this apoptotic response is attenuated, allowing resistant micrometastatic clones to persist after treatment, thereby increasing the likelihood of early recurrence [29].

The findings of this study have implications not only for understanding disease biology but also for informing clinical practice in patients with resected colorectal liver metastases. In patients who do not achieve a pathological complete response (pCR) after neoadjuvant FOLFOXIRI, aberrant p53 expression represents an independent prognostic factor that may facilitate postoperative risk stratification. These high‐risk patients should undergo more intensive surveillance using tumor markers and imaging to enable early detection of recurrence. Moreover, our results may support the personalization of adjuvant treatment strategies, such as considering more intensive chemotherapy regimens or targeted therapies, although further clinical studies are needed to validate the efficacy of such approaches. Finally, p53 immunohistochemistry is a simple, widely accessible, and cost‐effective technique that can be readily implemented in routine pathology practice as a tool for risk stratification.

In addition to recurrence‐free survival, we performed an exploratory analysis of overall survival. Aberrant p53 expression was not significantly associated with OS in our cohort (HR = 0.73, 95% CI: 0.16–3.27; *p* = 0.678). However, this finding should be interpreted with caution because only seven deaths occurred during follow‐up, limiting the statistical stability and reliability of the OS analysis. Therefore, RFS remained the more appropriate primary endpoint in the present study, as recurrence events were more frequent and more informative for evaluating early postoperative prognosis in this high‐risk population.

Although previous studies have suggested that concurrent TP53 and Ras/B‐raf pathway alterations may be associated with worse prognosis in colorectal cancer and colorectal liver metastases, we did not observe a statistically significant association in our cohort [30, 31]. This finding should be interpreted cautiously because only seven patients had concurrent aberrant p53 expression and RAS/BRAF alterations, which markedly limited the statistical power of this exploratory analysis. Therefore, our results do not exclude a possible combined prognostic effect but rather indicate that this question requires validation in larger cohorts with more comprehensive molecular profiling.

This study has several limitations. A key limitation of this study lies not merely in the absolute sample size (*n* = 39), but in the number of recurrence events relative to the number of potential prognostic variables. Given the constraints of event‐per‐variable recommendations for Cox proportional hazards modeling, we intentionally adopted a parsimonious multivariate approach to avoid model overfitting. As a result, molecular variables with relatively lower prevalence, such as KRAS/NRAS/BRAF mutations, could not be robustly evaluated for independent prognostic impact within the multivariate framework, despite being clinically relevant. This methodological choice prioritizes model validity over exploratory variable inclusion and should be interpreted accordingly. Second, the number of variables included in the multivariate model was restricted due to the need to balance sample size and model complexity to avoid overfitting. As a result, only variables with the lowest *p*‐values in univariate analysis were included, which may have limited the ability to control for clinically important confounders. Third, this study relied on p53 immunohistochemistry rather than molecular techniques to assess TP53 mutation status. Although the adoption of the updated pattern‐based interpretation improves the accuracy of IHC, there remains a potential for misclassification in inferring functional protein status [14, 32]. As this was a single‐center study conducted in an Asian population, caution is warranted when extrapolating these findings to other populations. Although the treatment protocol adhered to international standards and the biological mechanisms related to p53 function are not known to differ by ethnicity, external validation in multicenter cohorts is necessary to confirm the generalizability of these results.

## 5 Conclusion

Our study demonstrates that aberrant p53 expression, as assessed by immunohistochemistry, is an important independent prognostic factor for early recurrence in patients with colorectal liver metastases (CRLM) who did not achieve a pathological complete response (pCR) following neoadjuvant chemotherapy. Other independent predictors of shorter recurrence‐free survival (RFS) included total tumor size and positive resection margin. These findings enhance our understanding of recurrence risk, support risk stratification, inform postoperative surveillance strategies, and provide a rationale for personalized adjuvant treatment approaches aimed at improving clinical outcomes in this high‐risk population.

## Author Contributions

**Lam Viet Trung:** formal analysis. **Vuong Dinh Thy Hao:** investigation, writing – review and editing. **Thinh Van Hoang:** investigation. **Duy Duc Nguyen:** conceptualization, methodology, investigation, writing – original draft, writing – review and editing. **Phat Thi Hong Ho:** conceptualization, methodology, investigation, writing – original draft, writing – review and editing, project administration. **Hoang Bac Nguyen:** supervision, writing – review and editing. **Giang Huong Tran:** investigation.

## Funding

The authors have nothing to report.

## Ethics Statement

This study was approved by the Institutional Ethics Committee of Cho Ray Hospital (approval number: 1277/GCN‐HDDD, dated February 18, 2022).

## Consent

Written informed consent was obtained from all participants prior to inclusion in the study.

## Conflicts of Interest

The authors declare no conflicts of interest.

## Data Availability

The data that support the findings of this study are available on request from the corresponding author. The data are not publicly available due to privacy or ethical restrictions.
